# A combination of metformin and galantamine exhibits synergistic benefits in the treatment of sarcopenia

**DOI:** 10.1172/jci.insight.168787

**Published:** 2023-08-08

**Authors:** Caterina Tezze, Francesco Ivan Amendolagine, Leonardo Nogara, Martina Baraldo, Stefano Ciciliot, Diletta Arcidiacono, Alice Zaramella, Giulio Masiero, Giulia Ferrarese, Stefano Realdon, Bert Blaauw, Giel Detienne, Ann T.J. Beliën, Marco Sandri, Evi M. Mercken

**Affiliations:** 1 Department of Biomedical Sciences, University of Padova, Padova, Italy.; 2 Veneto Institute of Molecular Medicine, Padova, Italy.; 3 Gastroenterology Unit, Veneto Institute of Oncology IOV-IRCCS, Padova, Italy.; 4 Oncological Gastroenterology, Centro di Riferimento Oncologico di Aviano (CRO) IRCCS, Aviano, Italy.; 5 Rejuvenate Biomed, Diepenbeek, Belgium.; 6 Department of Medicine, McGill University, Montreal, Quebec, Canada.

**Keywords:** Aging, Muscle Biology, Drug therapy, Skeletal muscle

## Abstract

Age-associated sarcopenia, characterized by a progressive loss in muscle mass and strength, is the largest cause of frailty and disability in the elderly worldwide. Current treatments involve nonpharmacological guidelines that few subjects can abide by, highlighting the need for effective drugs. Preclinical models were employed to test the benefits of RJx-01, a combination drug composed of metformin and galantamine, on sarcopenia. In worms, RJx-01 treatment improved lifespan, locomotion, pharyngeal pumping, and muscle fiber organization. The synergistic effects of RJx-01 were recapitulated in a transgenic mouse model that displays an exacerbated aging phenotype (*Opa1^–/–^*). In these mice, RJx-01 ameliorated physical performance, muscle mass and force, neuromuscular junction stability, and systemic inflammation. RJx-01 also improved physical performance and muscle strength in 22-month-old WT mice and also improved skeletal muscle ultrastructure, mitochondrial morphology, autophagy, lysosomal function, and satellite cell content. Denervation and myofiber damage were decreased in RJx-01–treated animals compared with controls. RJx-01 improved muscle quality rather than quantity, indicating that the improvement in quality underlies the beneficial effects of the combination drug. The studies herein indicate synergistic beneficial effects of RJx-01 in the treatment of sarcopenia and support the pursuit of RJx-01 in a human clinical trial as a therapeutic intervention for sarcopenia.

## Introduction

During the aging process, there is a progressive loss of muscle strength and mass as well as an impairment in physical performance that are characteristic of sarcopenia and pose a threat to independent living. Sarcopenia, defined as an age-related loss in skeletal muscle mass and function, is one of the most pressing health problems in the elderly. Depending on the definition used for sarcopenia, the prevalence in 60- to 70-year-olds is reported to be between 6% and 22%, while the prevalence is as high as 50% in people > 80 years old (1). Additionally, sarcopenia is the largest cause of frailty and disability in the elderly around the world. Although the causes of sarcopenia are not completely understood, the underlying molecular mechanisms likely involve intrinsic factors — such as inflammation, mitochondrial dysfunction, denervation, oxidative stress, and extrinsic factors — like reduced calorie intake (e.g., low protein intake) and reduced physical activity (2, 3). Current treatment strategies for sarcopenia consist of nonpharmacological interventions such as lifestyle management, physical exercise, and adequate calorie and protein intake as first-line therapies (1). Not every subject, however, can abide by all of these recommendations; therefore, the therapeutic impact of the current interventions is limited, highlighting a medical need for medicinal products that prevent and/or treat sarcopenia.

To address the fact that there are no approved pharmacological agents for the treatment of sarcopenia, we tested the effectiveness of a combination drug, RJx-01 — a mixture of metformin (Met) and galantamine (Gal) — in multiple sarcopenia models. Met has been used for decades as the first-line treatment of type 2 diabetes (4). In the course of these treatments, Met has been revealed to have a beneficial effect on various age-related diseases in humans, with people with diabetes who take Met exhibiting a lower rate of all-cause mortality in comparison with nondiabetic people (5). Gal has been clinically approved and used for many years for the treatment of Alzheimer’s disease (AD) (6). Evidence from both human and animal studies indicates that Gal has health benefits that extend beyond cognition, implying a broader translational benefit of the drug (7). Similar to the situation with Met in patients with diabetes, treatment with Gal has been reported to significantly reduce all-cause mortality and improve functionality in patients with AD (8).

Published preclinical data indicate that Met and Gal elicit both distinct and overlapping effects on mechanisms relevant to the preservation of muscle strength and mass. In particular, studies indicate that Met targets a range of molecular pathways, with the AMP-activated protein kinase (AMPK) being a critical target. AMPK is known to increase mitochondrial biogenesis and oxidative metabolism (9), promote efficient autophagy (10), and stimulate muscle regeneration (11). Mitochondrial dysfunction and inflammation are 2 major factors that drive muscle aging or sarcopenia, and autophagy is an essential process required for maintaining skeletal muscle mass and whole-body energy metabolism (12). Thus, in targeting AMPK, Met appears to elicit a beneficial effect that counters skeletal muscle aging and sarcopenia. Gal, at the molecular level, operates via a dual mode of action — i.e., as an acetylcholinesterase (AChE) inhibitor and an allosteric positive modulator of nicotinic acetylcholine receptors (13). More generally, Gal exerts its beneficial effects by suppressing inflammation (14), a chronic condition in older adults that is considered a major contributor to sarcopenia, and exhibiting antioxidant properties, thereby minimizing oxidative damage, and a contributor to muscle decline. Due to its ability to promote efficient cholinergic neurotransmission (14), Gal potentially has a beneficial effect at the neuromuscular junction (NMJ), a highly specialized synapse between the motor neuron nerve terminal and its muscle fiber that facilitates muscle contraction.

Based on their different mechanisms of action, we questioned whether a combination of Met and Gal could have synergistic beneficial effects in treating sarcopenia. To test the benefits of RJx-01 on muscle performance and molecular mechanisms relevant to sarcopenia, we employed multiple preclinical animal models, first *Caenorhabditis elegans* and subsequently 2 mouse models: an inducible muscle-specific optic atrophy 1–KO (OPA1-KO) mouse model and aged mice. The *Opa1^–/–^* mouse model is a well-known model that recapitulates key pathophysiological aspects of sarcopenia-related muscle loss and strength (15).

## Results

### RJx-01 synergistically extends lifespan and improves fitness in C. elegans.

As a first test of the effectiveness of RJx-01, we evaluated the potential benefits of the combination drug on a series of endpoints related to lifespan and fitness in *C*. *elegans*. *C*. *elegans* is considered a relevant nonclinical model for investigating the processes related to aging and a tool for drug discovery (16). Important to the study here, the loss of muscle mass and function with aging in *C*. *elegans* shows a similar progression to sarcopenia in humans (17). Met is well described for prolongevity effects in *C*. *elegans* (18), yet no lifespan studies have been performed using Gal. Additionally, the effects of their combination at suboptimal doses have not been explored. We performed a dose range study for the individual compounds and evaluated the potential benefits of Met and Gal in combination. The combination, 25 mM Met + 100 μM Gal, was selected based on their synergistic effects on lifespan and fitness.

**Figure 1:**
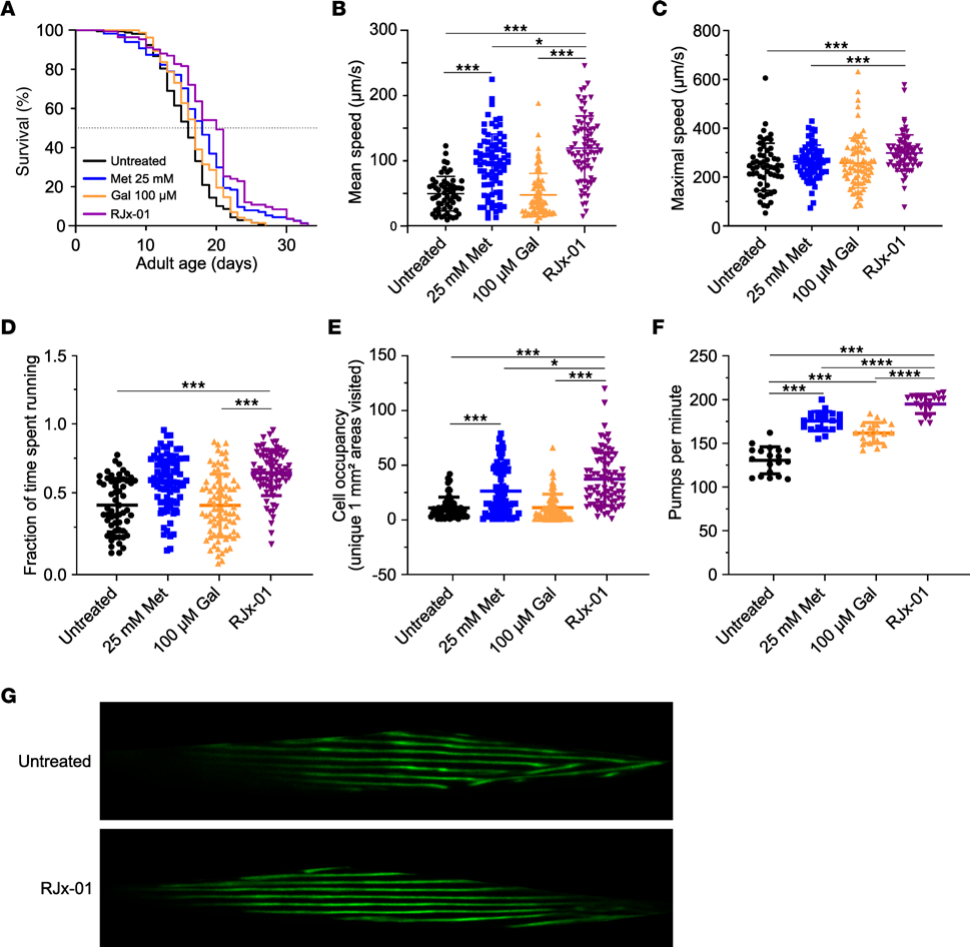
RJx-01 synergistically extends lifespan and improves fitness in *C*. *elegans*. (**A**) The manual lifespan of worms treated with 25 mM Met, 100 μM Gal, the combination drug RJx-01 (25 mM Met and 100 μM of Gal), or with vehicle (water). For additional data, see Supplemental Table 3. (**B**–**E**) Locomotion of worms at day 7 of adulthood in worms treated with 25 mM Met, 100 μM Gal, the combination drug RJx-01, or vehicle (*n* = 60–80 per group). (**F**) Pharyngeal pumping at day 7 of adulthood in worms treated with 25 mM Met, 100 μM Gal, the combination drug RJx-01, or vehicle (water) (*n* = 10). (**G**) Representative images of muscle morphology at day 14 of adulthood of RW1596 worms treated with the combination drug RJx-01 or vehicle. Scale bar: 10 μm. Data are representative of at least 3 independent experiments. Data are shown as mean ± SEM. *P < 0.05; ***P < 0.001; ****P < 0.0001. In **A**, Kaplan-Meier curves were generated, and statistical significance was calculated using the log-rank test followed by Benjamini-Hochberg. One-way ANOVA followed by Holm step-down method (2-tailed Student’s *t* test) was performed in **B**–**F**.

To characterize the beneficial effect of this drug combination (named RJx-01) and its individual components (25 mM Met and 100 μM Gal) in *C*. *elegans*, we performed a series of experiments on solid agar medium. In line with previous work (18), 25 mM Met resulted in an increased lifespan compared with untreated animals (+11.9% mean lifespan; Figure 1A and Supplemental Table 3; supplemental material available online with this article; https://doi.org/10.1172/jci.insight.168787DS1). A significantly increased lifespan was also observed with Gal (+6.8% mean lifespan; Figure 1A and Supplemental Table 3), albeit to a lesser extent than Met. The combination drug, RJx-01, induced the strongest lifespan extension, with a mean lifespan increase of +22.1% relative to the untreated control, an effect that was significantly greater than the individual components.

We next evaluated the effect of RJx-01 on worm activity by assessing multiple locomotion parameters and pharyngeal pumping rates. In all cases, the highest level for mean speed, maximal speed, the fraction of time spent running, and cell occupancy was attained in animals treated with RJx-01 (Figure 1, B–E). RJx-01 significantly increased maximal speed (by 24%), mean locomotion speed (by 141%), the fraction of time spent running (by 46%), and cell occupancy (by 239%) compared with the untreated control. In line with the synergistic effect of RJx-01 on locomotion, treatment with the combination drug resulted in a synergistically improved pharyngeal pumping rate relative to the individual compounds alone (Figure 1F). This improved fitness in the RJx-01–treated group correlated with better maintenance of the muscle fiber organization (Figure 1G), indicating the beneficial effects of the combination drug on muscle structure and function.

### RJx-01 treatment suppresses muscle mass and quality loss and enhances functional outcomes in Opa1^–/–^ mice.

To extend our initial observations in worms to mammals, we tested the efficacy of RJx-01 in a recently developed mouse model that, by inducing mitochondrial dysfunction specifically in skeletal muscles, exhibits a precocious aging phenotype. This mouse line — i.e., the inducible muscle-specific Opa1-KO *—* mimics the age-dependent decline of the fusion protein OPA1 in skeletal muscles of patients and mice with sarcopenia. Indeed, acute inhibition of OPA1 in adult animals led to a geriatric phenotype with the classical features of sarcopenia such as muscle mass and strength loss, denervation, and exercise intolerance in just 3 months. Moreover, these mice showed a generalized aging phenotype characterized by a systemic sterile inflammatory response in blood, cellular senescence of epithelial tissues, and premature death (15). Thus, this genetic model looks sufficient to validate the effects of RJx-01 on sarcopenia in mammals.

**Figure 2:**
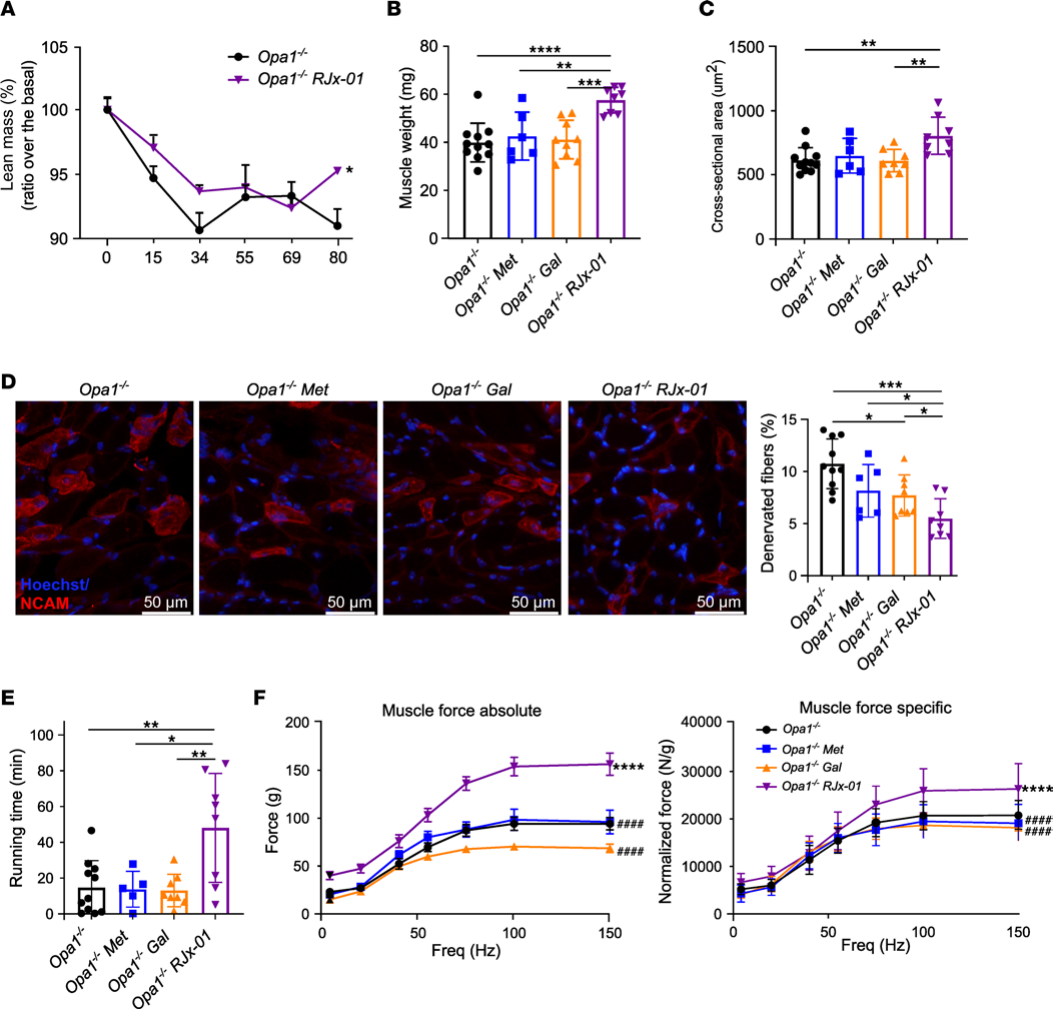
RJx-01 treatment suppresses muscle mass and quality loss and enhances functional outcomes in muscle-specific *Opa1^–/–^* mice. (**A**) Percentage of lean mass of *Opa1^–/–^* mice fed a control diet or a diet supplemented with RJx-01 (*Opa1^–^/^–^*, *n* = 11; *Opa1^–/–^ RJx-01*, *n* = 8). (**B**) Weights of gastrocnemius muscles of *Opa1^–/–^* mice were fed a control diet, a control diet supplemented with metformin (Met), a control diet supplemented with galantamine (Gal), and a control diet supplemented with RJx-01 (*Opa1^–^/^–^*, *n* = 11; *Opa1^–/–^ Met*, *n* = 6; *Opa1^–/–^ Gal*, *n* = 9; *Opa1^–/–^ RJx-01*, *n* = 8). (**C**) Myofibers cross-sectional area analysis (*Opa1^–^/^–^*, n=11; *Opa1^–/–^ Met*, *n* = 6; *Opa1^–/–^ Gal*, *n* = 9; *Opa1^–/–^ RJx-01*, *n* = 8). Scale bar: 50 μm. (**D**) Representative images of immunostaining for NCAM expression, and quantification of denervated NCAM^+^ fibers in the respective groups (*Opa1^–^/^–^*, *n* = 10; *Opa1^–/–^ Met*, *n* = 6; *Opa1^–/–^ Gal*, *n* = 8; *Opa1^–/–^ RJx-01*, *n* = 8). (**E**) Exercise performance on the treadmill expressed as running time (*Opa1^–^/^–^*, *n* = 11; *Opa1^–/–^ Met*, *n* = 5; *Opa1^–/–^ Gal*, *n* = 8; *Opa1^–/–^ RJx-01*, *n* = 8). (**F**) Force-frequency curves were performed in vivo on gastrocnemius muscles. Absolute force and maximal specific force generated during tetanic contraction in the respective groups (*Opa1^–^/^–^*, *n* = 6; *Opa1^–/–^ Met*, *n* = 3; *Opa1^–/–^ Gal*, *n* = 5; *Opa1^–/–^ RJx-01*, *n* = 5). Data are shown as mean ± SEM. *P < 0.05; **P < 0.01; ***P < 0.001; ****P < 0.0001; ^####^*P* < 0.0001 compared with *Opa1^–/–^* RJx-01. In **A** and **F**, a linear mixed-effects model was used, and 1-way ANOVA followed by Holm step-down method (2-tailed Student’s *t* test) was performed in **B**–**E**.

After acute deletion of Opa1 in adulthood, the *Opa1^–/–^* animals were divided into 4 groups: a control group (untreated), a group treated with Met (410 mg/kg bodyweight/d), a group treated with Gal (3.28 mg/kg bodyweight/d), and a group treated with RJx-01 (combination of 410 mg/kg bodyweight/d Met and 3.28 mg/kg bodyweight/d Gal) (Supplemental Figure 1J). As shown in Supplemental Figure 1A, RJx-01 partially reduced body weight loss in *Opa1^–/–^* mice compared with untreated *Opa1^–/–^* mice. Moreover, RJx-01 prevented the lean mass loss in *Opa1^–/–^* mice (Figure 2A), with no difference in fat mass (Supplemental Figure 1B). The individual compound Met did not have an effect on body weight, lean mass, or fat mass (Supplemental Figure 1, C–E), whereas Gal slightly prevented lean and fat mass loss (Supplemental Figure 1, F–H). Importantly, the differences in body composition were not due to altered food intake (Supplemental Figure 1I) but were the result of RJx-01 treatment.

Consistent with the maintenance of lean mass, tibialis anterior (Supplemental Figure 1K), and gastrocnemius (Figure 2B) muscle mass and the cross-sectional area (CSA) of myofibers (Figure 2C) were significantly increased in the RJx-01–treated animals compared with the untreated or single-compound–treated mice. This was confirmed by quantifying the average minimal Feret’s diameter (Supplemental Figure 1L). During aging, a progressive decline in lean muscle mass can be associated with the preferential loss of glycolytic, fast-twitch myofibers (19), which promotes the decrease of the gait speed in humans. Importantly, RJx-01–treated mice maintained a higher abundance of fast glycolytic fibers (Supplemental Figure 1, M–O) than untreated and Gal-treated *Opa1^–/–^* mice.

The decrease in fast fibers is caused by the loss of fast motor neurons and myofiber innervation (3). Indeed, when we tested myofiber denervation by neural cell adhesion molecule (NCAM) staining, we found that RJx-01 treatment significantly reduced by half the number of denervated fibers when compared with untreated and individual compound–treated animals (Figure 2D and Supplemental Figure 1P).

The decrease in innervation and the loss of fast fibers negatively impact functional outcomes in general and force generation and physical performance specifically. Indeed, *Opa1^–/–^* mice are weak like geriatric mice, and they run on a treadmill at very low speed and for a short time. Importantly, RJx-01 treatment significantly increased muscle performance with a 3-fold increase in running time compared with the controls (Figure 2E). Consistently, muscle strength was greatly improved in both the absolute (+66%) and specific (+38%) force (Figure 2F). The increase in specific force further sustains the beneficial effect of the treatment on myofiber innervation. These results collectively indicate that RJx-01, but not Met or Gal alone, counteracts atrophy, denervation, weakness, and exercise intolerance in a genetic model of precocious aging.

### RJx-01 treatment reduces systemic and muscular inflammatory markers in Opa1^–/–^ mice.

**Figure 3:**
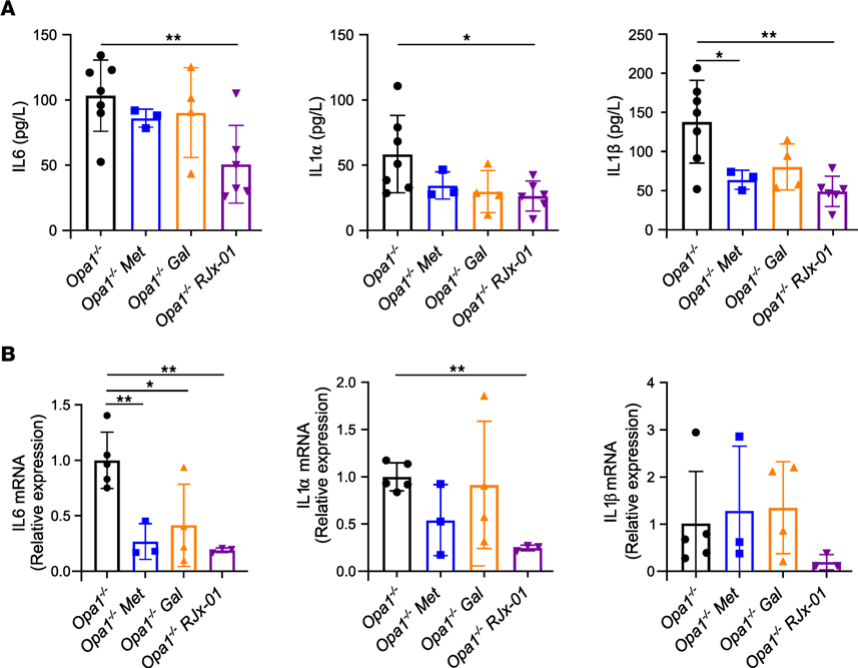
RJx-01 treatment reduces systemic and muscular inflammatory levels. (**A** and **B**) Inflammatory (IL-6, IL-1α, and IL-1β) levels in serum (*Opa1^–^/^–^*, *n* = 7; *Opa1^–/–^ Met*, *n* = 3; *Opa1^–/–^ Gal*, *n* = 4; *Opa1^–/–^ RJx-01*, *n* = 6) (**A**) and tibialis anterior muscles of *Opa1^–/–^* mice (**B**) fed a control diet or a diet supplemented with metformin (Met), a control diet supplemented with galantamine (Gal), a control diet supplemented with RJx-01 (*Opa1^–/–^*, *n* = 5; *Opa1^–/–^ Met*, *n* = 3; *Opa1^–/–^ Gal*, *n* = 4; *Opa1^–/–^ RJx-01*, *n* = 3). Data are shown as mean ± SEM. *P < 0.05; **P < 0.01 by 1-way ANOVA followed by Holm step-down method (2-tailed Student’s *t* test).

At the systemic level, chronic, low-grade “sterile” inflammation is considered a hallmark of aging and is associated with frailty syndrome, the reduced capacity to respond to stress (20). *Opa1^–/–^* mice are characterized by a more than 10-fold increase of inflammatory cytokines (e.g., IL-6, IL-1α, IL-1β, TNF-α) in the blood (15). Interestingly, RJx-01 treatment reduced plasma levels of IL-6, IL-1α, and IL-1β when compared with the untreated mice (Figure 3A). Consistent with the blood levels, the upregulation of these cytokines was totally or partially blunted in the skeletal muscles of the RJx-01–treated mice (Figure 3B).

### RJx-01 increases physical performance and strength and reduces denervation in the skeletal muscle of aged mice.

**Figure 4:**
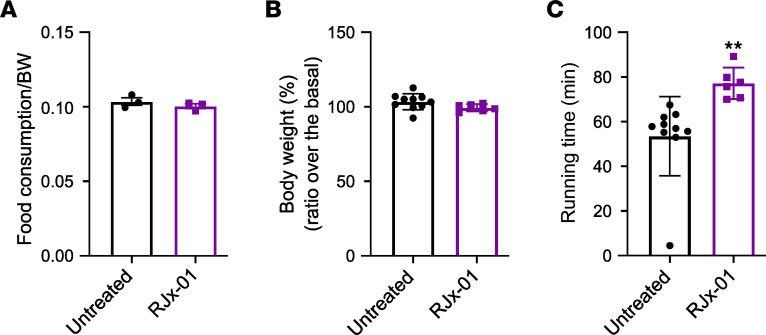
RJx-01 increases physical performance, without modifying food intake and body weight in aged mice. (**A**) Food consumption of aged mice fed a control diet (untreated) and a diet supplemented with RJx-01 (*n* = 3 cages, 5 mice per cage). (**B**) Percentage body weight expressed as a ratio over basal body weight (Untreated, *n* = 10; RJx-01, *n* = 6). (**C**) Running time following 18 weeks treatment (Untreated, *n* = 10; RJx-01, *n* = 6). Data are shown as mean ± SEM. **P < 0.01 by 2-tailed Student’s *t* test.

The findings in worms and the transgenic mice support the beneficial and synergistic effect of Met and Gal in counteracting sarcopenia. To further corroborate these data, we moved to WT aged mice. Since only the combination of the drugs maximized the beneficial effects, we treated 22-month-old mice with only RJx-01 (Supplemental Figure 2A) for 18 weeks and monitored muscle mass, exercise tolerance, and force generation. Food intake (Figure 4A) and body weight (Figure 4B) were not affected by the treatment, but physical activity monitored by the running time was significantly improved when compared with controls (Figure 4C). This effect was observed despite muscle mass, mean fiber size, fiber size distribution, and fiber type being similar among the 2 groups (Supplemental Figure 2, B–F). These findings suggest that RJx-01 improves muscle function more than muscle mass.

**Figure 5:**
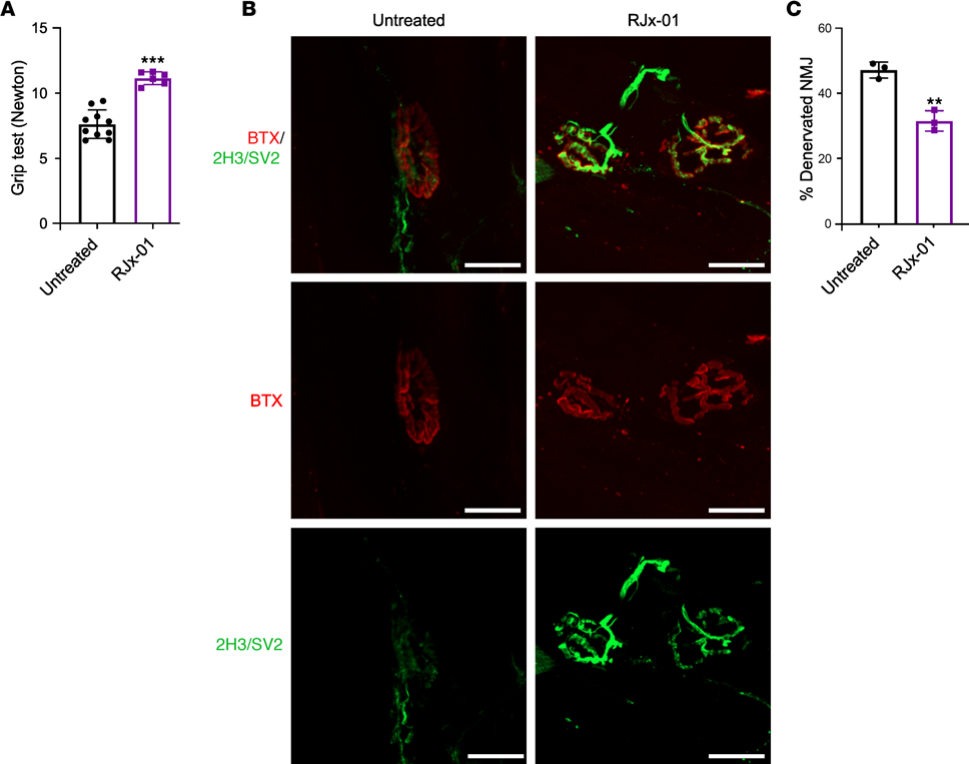
RJx-01 increases muscle strength and reduces the number of denervated fibers in skeletal muscle of aged mice. (**A**) Grip test in aged mice treated with and without RJx-01 for 18 weeks (Untreated, *n* = 10; RJx-01, *n* = 6). (**B**) Labeled presynaptic (neurofilament [2H3]; synaptic vesicle 2 [SV2]; green) and postsynaptic (bungarotoxin [BTX]; red) NMJ components in EDL from aged mice supplemented with and without RJx-01 for 18 weeks. Scale bar: 20 μm. At least 200 NMJs were analyzed per mouse. (**C**) Percentage of denervated fibers in the EDL muscle of untreated and RJx-01–treated mice (*n* = 3 per group). Data are shown as mean ± SEM. **P < 0.01; ***P < 0.001 by 2-tailed Student’s *t* test.

A progressive loss of strength is commonly observed with aging, and in humans, handgrip strength is considered an indicator of overall muscle strength and is used to diagnose sarcopenia (21). We used a similar test for assessing mouse muscle strength, measuring the amount of static force that can be generated around a dynamometer. Mice treated with RJx-01 showed a 32% increase in grip strength compared with the control group (Figure 5A).

Since muscle mass and fiber size were not affected by the treatment, while force generation and physical activity were improved, we reasoned that stability and morphology of muscle-nerve synapses might be affected (3). Indeed, rodents experience a loss of innervation during aging, an event that occurs before myofiber atrophy (22). Moreover, the reinnervation process of denervated fibers is impaired in old mice (23). Therefore, we monitored NMJ morphology by staining the postsynaptic side of NMJ with α-bungarotoxin (αBTX) and the presynaptic side with synaptic vesicle 2 (Sv2) and monitored how many fibers of whole EDL muscle did not show a match between the 2 markers (denervated fibers). RJx-01–treated mice showed a better morphology of NMJ with a good overlap between the post- and presynaptic sides (Figure 5B) that resulted in 15% less denervated fibers compared with untreated mice (Figure 5C).

### RJx-01 protects the skeletal muscle ultrastructure and mitochondrial morphology in aged mice.

**Figure 6:**
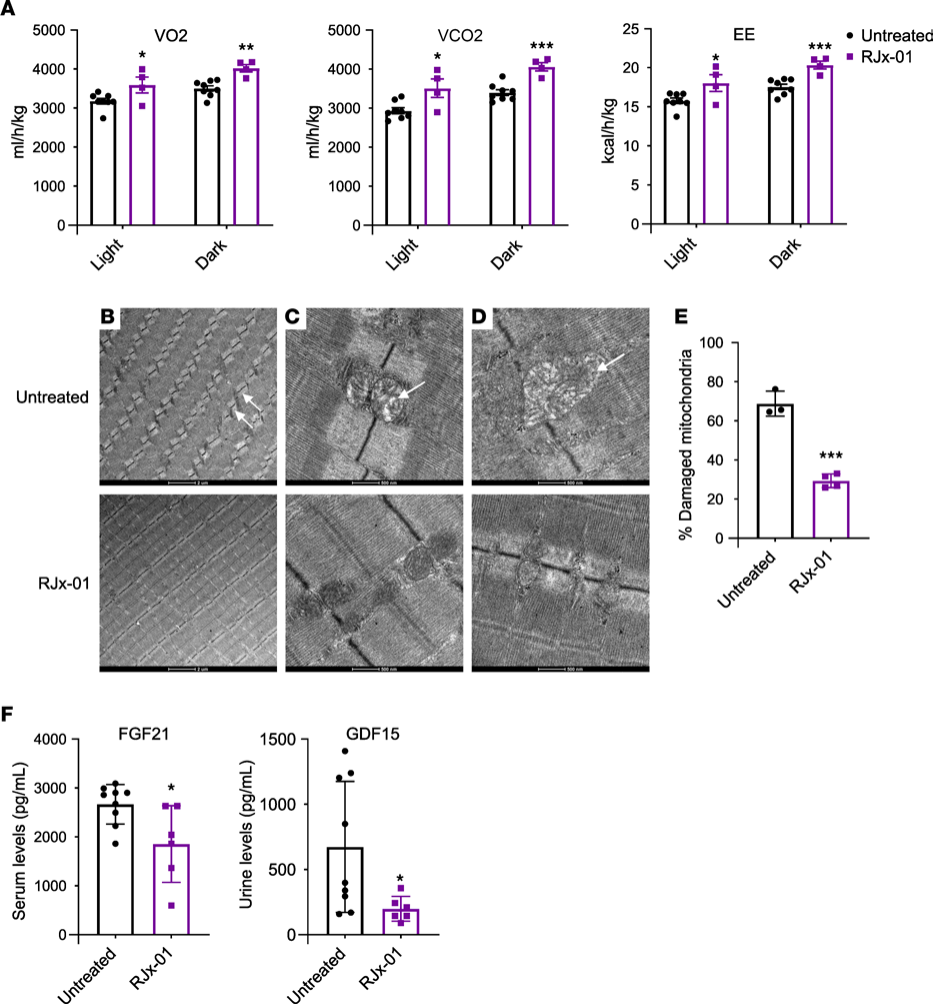
RJx-01 protects the skeletal muscle ultrastructure and mitochondrial morphology in aged mice. (**A**) Oxygen consumption (VO_2_), carbon dioxide production (VCO_2_), and energy expenditure (EE) in aged mice treated with RJx-01 for 10 weeks (Untreated, *n* = 8; RJx-01, *n* = 4). (**B**–**D**) Representative electron micrographs of EDL muscles of aged mice treated with and without RJx-01. (**B**) Arrows represent sarcomere misalignment. Scale bar: 2 µm. (**C**) The arrow denotes less electron-dense matrix mitochondria. (**D**) The arrow points to a swollen mitochondrion. Different magnifications are shown. Scale bars: 500 nm. (**E**) Number of damaged mitochondria in EDL muscle of untreated and RJx-01–treated mice (number of mitochondria analyzed per mouse, 209–288) (Untreated, *n* = 3; RJx-01, *n* = 4). (**F**) Serum FGF21 and urine GDF15 levels (Untreated, *n* = 9; RJx-01, *n* = 6). Data are shown as mean ± SEM. *P < 0.05; **P < 0.01; ***P < 0.001 by 2-tailed Student’s *t* test.

Besides the positive action on NMJ, the improvement of muscle function by RJx-01 may also result from a beneficial effect on the mitochondrial network and bioenergetics. Energy expenditure (EE) — as well as oxygen consumption (VO_2_) and carbon dioxide production (VCO_2_) — are known to decrease with advanced age in mice and humans (24). Mice housed in metabolic cages and treated with RJx-01 showed higher scores for all metabolic parameters (VO_2_, VCO_2_, and EE) than controls (Figure 6A). Because of the improved muscle function and the positive effect on EE, we looked at the effect of RJx-01 on mitochondrial morphology. Indeed, mitochondrial dysfunction and reduced mitochondrial quality control are recognized contributors to the pathogenesis of sarcopenia, and mitochondria have been reported to be fragmented or atypically enlarged in skeletal muscle from aged rodents or humans (25). Ultrastructural analyses at electron microscopy (EM) of EDL muscles showed several abnormalities of the sarcomere structure and mitochondrial network in untreated aged mice. Consistent with the literature, sarcomeres of aged mice displayed Z-line misalignment (Figure 6B, top panel), a characteristic that was reversed by RJx-01 treatment (Figure 6B, bottom panel). Importantly, the mitochondria of the untreated aged mice showed a reduced number of cristae, an electron-pale matrix (Figure 6C, top panel), and a swollen appearance (Figure 6D, top panel) — all features of damaged mitochondria. RJx-01 treatment ameliorated mitochondrial morphology (Figure 6, C and D, bottom panel) and reduced the number of damaged mitochondria by half (Figure 6E).

Because mitochondrial dysfunction triggers the expression and the secretion of mitokines such as FGF21 and GDF15 from muscles (15, 26), we checked the blood and urine levels of these cytokines, respectively, as a readout of mitochondrial functionality. Blood FGF21 and urine GDF15 levels were significantly decreased in RJx-01–treated animals when compared with the untreated controls (Figure 6F), suggesting improved mitochondrial activity.

### RJx-01 ameliorates autophagy and lysosomal function in aged muscles.

**Figure 7:**
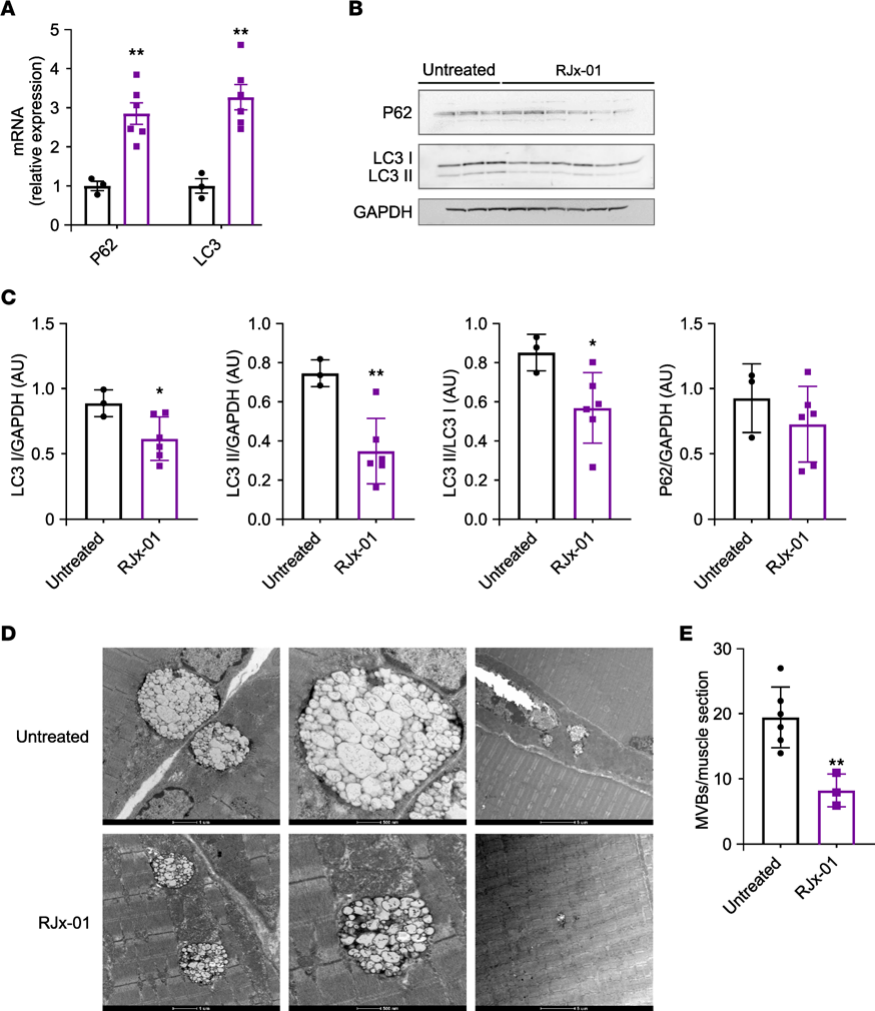
RJx-01 increases autophagy in the skeletal muscle of aged mice. (**A**) qPCR analysis of transcriptional levels relative to the target genes involved in the autophagic process (Untreated, *n* = 3; RJx-01, *n* = 6). (**B**) Representative Western blot of LC3I/II, P62, and GAPDH of whole protein muscle extract. (**C**) The relative quantification of **B** (Untreated, *n* = 3; RJx-01, *n* = 6). (**D**) Representative electron micrographs of EDL muscles of aged mice treated with and without RJx-01. Representative image of an enlarged multivesicular body (MVB). Different magnifications are shown, and scale bar sizes are indicated. Scale bar: 1 µm (left image); 500 nm (middle and right images). (**E**) Number of MVBs in the muscle of untreated and RJx-01–treated mice (Untreated, *n* = 6; RJx-01, *n* = 6). Data are shown as mean ± SEM. *P < 0.05, **P < 0.01 by 2-tailed Student’s *t* test.

Autophagy is an important player in mitochondrial quality control and myofiber integrity. Moreover, we have shown that autophagy inhibition in muscles induces mitochondrial dysfunction, oxidative stress, myofiber denervation, muscle loss, force drop, and premature death (27). Finally, we and others have previously shown that autophagy impairment occurs in aged mice and elderly people (27, 28). Thus, we tested whether RJx-01 treatment reactivates autophagy in old mice by measuring P62 and LC3 changes. RJx-01 administration increased P62 and LC3 transcripts (Figure 7A). Despite the transcript upregulation, both the lipidated and not lipidated LC3 were decreased, while no changes were observed in the P62 protein level (Figure 7, B and C, and Supplemental Figure 3). The transcriptional upregulation of LC3 and P62 indicates that autophagy is transcriptionally enhanced, but the decrease of LC3 protein and the lack of P62 accumulation suggests an increased degradation that compensates for the transcriptional upregulation and, therefore, an autophagy flux restoration. Autophagy impairment in aging is also consequent to a lysosomal failure. In fact, EM analysis of aged muscles has shown dysfunction of the autophagosome-amphisome-lysosome system revealed by the accumulation in perinuclear regions of autophagolysosomes that are positive for LAMP1 and enriched in lipofuscin (29). These autophagolysosome features contain undigested material resembling huge multivesicular bodies (MVBs) (Figure 7D). We quantified these structures as a morphological index of lysosomal impairment and found that RJx-01 treatment significantly reduced them when compared with untreated controls (Figure 7E). In addition, because these structures are LAMP1^+^, we immunostained the muscles for LAMP1 and found that RJx-01 treatment reduced LAMP1^+^ puncta (Supplemental Figure 3A).

### RJx-01 reduces myofiber damage and increases the number of Pax7^+^ satellite cells in the skeletal muscle of aged mice.

**Figure 8:**
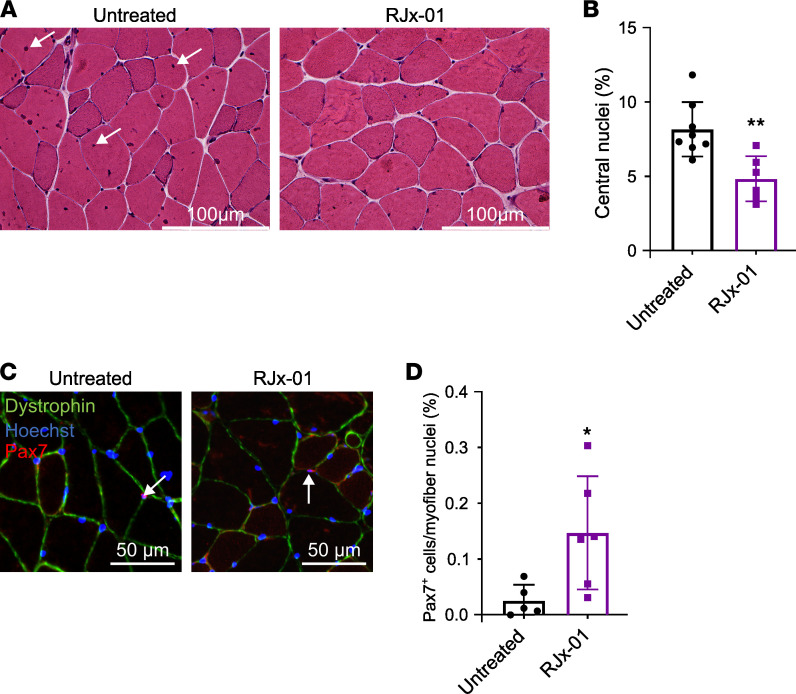
RJx-01 reduces the damage in myofibers and increases the number of Pax7^+^ satellite cells in the skeletal muscle of aged mice. (**A**) Representative H&E images of the gastrocnemius cross-sections. Arrows show central nuclei. Scale bar: 100 μm. (**B**) Quantification of myofibers with central nuclei in gastrocnemius muscles as a percentage of total myofibers (Untreated, *n* = 8; RJx-01, *n* = 6). (**C**) Representative images of Pax7 staining. Scale bar: 50 μm. Arrows indicate the nuclei positive for Pax7. (**D**) Quantification of the percentage of Pax7^+^ cells in the gastrocnemius skeletal muscle of old mice treated with and without RJx-01 (Untreated, *n* = 5; RJx-01, *n* = 6). Data are shown as mean ± SEM. *P < 0.05; **P < 0.01 by 2-tailed Student’s *t* test.

We have shown that depletion of muscle stem cells occurs in elderly mice because autophagy impairment blocks stem cell renewal (30). Age-related cell stress predisposes muscle fiber damage that necessitates repair/regeneration events; therefore, the stem cell renewal, differentiation, and fusion events are more frequent, leading to an increase of centrally nucleated fibers. Thus, the number of muscle stem cells available for repair and their ability to expand upon injury gradually declines with age (31). The inability of muscle stem cells to repair damaged muscle fibers potentially contributes to the functional decline observed in the elderly (32). As a result, the recovery from muscle injuries caused by falls or other traumatic events is considerably lower in aged individuals. Since muscle regeneration or stem cell fusion is revealed by the presence of centrally nucleated fibers, we quantified the centrally nucleated fibers. RJx-01 treatment significantly reduced the number of central nuclei when compared with controls (Figure 8, A and B). The reduction of these fibers may arise by the preservation of the muscle stem cell or by reducing the fiber damage and regeneration. To identify whether the first option is true, we monitored the transcription factor Pax7, which is the marker of the muscle stem cells named satellite cells. Aged mice treated with RJx-01 displayed a significant increase in the percentage of Pax7^+^ cells when compared with the untreated mice (Figure 8, C and D), supporting the concept that the drug improved muscle stem cell renewal and maintenance.

## Discussion

The functional deterioration of skeletal muscle with age is one of the major causes of loss of independence, nursing home admission, development of comorbidities, and increased mortality in the elderly. Considering the high costs associated with the care of these patients, sarcopenia and frailty are an enormous burden on healthcare systems. Based on the expanding global geriatric population, effective interventions to preserve or improve physical performance and functional capacities at advanced ages are, thus, becoming increasingly important, on both the individual and societal levels. We present preclinical evidence in 2 model organisms that the combination drug, RJx-01— composed of Met and Gal — exhibits synergistic benefits in the treatment of sarcopenia.

First, we used the classic agar platform to examine the efficacy of RJx-01 on lifespan and fitness (movement) in *C*. *elegans*. Our studies reveal that the combination drug shows a clear synergistic effect in prolonging survival, increasing the quality and speed of movements, and improving muscle structures compared with the individual compound treatments. Moreover, we confirmed the effect of RJx-01 in an inducible muscle-specific OPA1-KO mouse model, which recapitulates in just 3 months all the features of sarcopenia (e.g., atrophy, weakness, reduced physical activity, fiber type switching, mitochondrial dysfunction, oxidative stress, denervation, and a sterile systemic inflammatory response). Upon treatment with RJx-01, *Opa1^–/–^* mice showed improved muscle force and performance that was not observed after treatment with Met or Gal alone. These results indicate that RJx-01 is effective at preserving muscle function, reducing exercise intolerance and weakness in a genetic model of precocious aging, and that drug efficacy involves synergy between Met and Gal. At the muscle level, RJx-01 reduced denervation in *Opa1^–/–^* mice maintaining fast fibers, while at the systemic level, RJx-01 administration reduced plasma levels of inflammatory markers, IL-6, IL-1α, and IL-1β. Systemic chronic inflammation is an important contributor to aging and a key player in promoting sarcopenia and frailty, decreasing muscle strength, and impairing the stress response (20). Thus, the findings in worm and *Opa1^–/–^* mice argue for a synergistic beneficial effect of RJx-01 in the treatment of sarcopenia.

Using 22-month-old mice, we observed that RJx-01 ameliorated grip strength, in part by preserving the NMJ and in part by ameliorating bioenergetics and mitochondrial network. Specifically, RJx-01–treated mice were 32% stronger and showed 15% more innervated muscles than controls. We also found that EE increased with RJx-01 treatment, a phenotype that did not coincide with increased muscle mass. Since EE, VO_2_, and VCO_2_ normally decrease in humans and mice with advanced age (24), the improved metabolism following RJx-01 treatment is due to improved mitochondrial morphology. Ultrastructural analysis by EM of aged EDL muscles from RJx-01–treated mice revealed a reduction in mitochondrial abnormalities when compared with the untreated samples. The autophagic process, which decreases with age and contributes to mitochondrial dysfunction and the onset of sarcopenia, was found to be improved after 18 weeks of RJx-01 treatment. Since autophagy inhibition has been found to promote denervation, its improvement may explain the preservation of NMJ stability and mitochondrial health.

Previously, we have shown that autophagy impairment causes an increase in centrally nucleated fibers and a depletion of muscle stem cells (30). Moreover, prior studies have shown that autophagy has an important role in stem cell maintenance (30). In line with the amelioration of autophagy and lysosomal trafficking upon RJx-01 treatment, we observed a significant reduction in centrally nucleated fibers and increased preservation of the satellite cell reservoir. This is important because, with age, muscle stem cells are unable to repair damaged muscle fibers, lowering the capacity of muscle growth during the reloading phase (32). Since the amount of muscle stem cells that are available to recover after muscle injury declines with age (33), our data suggest that RJx-01 treatment might counteract injury-induced sarcopenia by preserving the muscle stem cell pool.

In conclusion, we show the beneficial effects of a Met and Gal combination drug (RJx-01) as a treatment agent in sarcopenia. In particular, RJx-01 synergistically enhanced muscle strength and improved running endurance, without a consistent effect on muscle mass. RJx-01 improved muscle cell quality (e.g., reducing inflammation, increasing NMJ stability, preserving mitochondrial health, enhancing autophagy and lysosomal trafficking, and supporting stem cell reservoir maintenance) rather than muscle quantity, indicating that the improvement in quality by RJx-01 underlies its beneficial effects. The data herein support the pursuit of RJx-01 in human clinical trials as a therapeutic intervention for sarcopenia.

## Methods

*C. elegans* strains and pharmacological treatment

Bristol N2 WT and RW1596 (myo-3[st386];myo-3p:MYO-3:GFP+rol-6[su1006]) strain (34) were obtained from the Caenorhabditis Genetic Center (University of Minnesota, Minneapolis, Minnesota, USA) and the Braekman laboratory (Ghent University, Ghent, Belgium), respectively, and were cultured using standard methods at 20°C on standard nematode growth medium (NMG) seeded with OP50 *E*. *coli* bacteria as a food source unless otherwise noted. Gal hydrobromide (PHR1623) and 1,1-Dimethylbiguanide hydrochloride (D150959) were purchased from Sigma-Aldrich.

### Culturing and treatment of the worm strain on agar

The WT *C*. *elegans* N2 strain was cultivated at 20°C on NGM seeded with a thin layer of *E. coli* OP50. Met was added at the indicated concentration to the *C*. *elegans* culture medium mixture, before autoclaving. Gal stock solutions were prepared in ultrapure Milli-Q water and were sterile filtered, before administeriion to *C*. *elegans* agar plates. Worms were exposed to the compounds from the late L4 stage until death unless stated otherwise. To ensure a permanent exposure to the compound, plates were changed 2 or 3 times a week. Both compounds were dissolved in ultrapure Milli-Q stock solution, and an equal volume of water was added to the respective controls.

#### Lifespan.

Lifespan experiments were performed as previously described (35). To ensure permanent exposure to the compound, worms were transferred 3 times in the first week and weekly thereafter (i.e., on days 2, 4, 7, 15, 22, and 29). Worms that crawled off the plate or died of vulval bursting or internal hatching were censored.

### Worm phenotypic assays

#### Mobility.

*C*. *elegans* movement was performed on day 7 of adulthood, using a custom particle-tracking MATLAB code as previously described (36, 37). Movement consisted of mean and maximum speed, the fraction of time spent running (fraction of time that an animal was detected to be running), and cell occupancy (the number of unique cells that the worm visited during the imaging period). Off-food locomotion was assessed for which around 20 well-fed worms were picked to an unseeded NGM plate and then, within 5 minutes, transferred to a second unseeded NGM plate used for imaging; no residual OP50 was present on the plates. Experiments were performed in triplicate.

#### Pharyngeal pumping.

Pharyngeal pumping rates were quantified using a stereomicroscope, according to the guidelines described (38). Before the start of the assay, at least 10 individual animals per condition were manually transferred to distinct NGM plates seeded with a thin layer of OP50. After 7 days of adulthood, the pharyngeal pumping was measured by counting the contraction of the pharynx during 2 distinct 30-second periods.

### Mouse models

#### Opa1^–/–^ mice.

The generation and characterization of the inducible muscle-specific *Opa1^–/–^* mice is described in detail elsewhere (15). Mice were fed a standard house chow (4RF21, Standard Diet Certificate from Mucedola S.R.L.), a standard house chow supplemented with 3.0 g Met/kg chow or 23.8 mg Gal/kg chow, or RJx-01 (3.0 g Met/kg chow and 23.8 mg Gal/kg chow). Taking into consideration body weight and food consumption, the chow was formulated to provide a daily dose of 410 mg Met/kg of body weight, or a daily dose of 3.28 mg Gal/kg of body weight, or the combination of both compounds (RJx-01). The individual compounds, Met (410 mg/kg bodyweight/d) or Gal (3.28 mg/kg bodyweight/d), or RJx-01 (410 mg/kg bodyweight/d Met and 3.28 mg/kg bodyweight/d Gal) were admixed to house chow. Pellets with or without the individual compounds or RJx-01 were given after tamoxifen treatment to male *Opa1^–/–^* mice for a maximum of 90 days. Muscle and blood samples were collected at sacrifice and the numbers of animals sacrificed after treatment are: *Opa1^–/–^*, *n* = 11; *Opa1^–/–^* Met, *n* = 6; *Opa1^–/–^* Gal, *n* = 9; *Opa1^–/–^* RJx-01, *n* = 8.

#### Aged mice.

Thirty 22-month-old male C57BL/6JRj mice (Janvier Labs) were randomly assigned to one of the 2 groups following 18 weeks of intervention (*n* = 15 per group); mice were fed a standard house chow (4RF21, Standard Diet Certificate from Mucedola S.R.L.) or a standard house chow formulated so mice received a daily dose of 410 mg Met/kg of body weight and 3.28 mg Gal/kg of body weight (RJx-01 group). Mice were provided water and food ad libitum. Due to the COVID-19 lockdown in Italy, only a subset of animals was sacrificed after 12 weeks. The numbers of animals sacrificed after 12 weeks of treatment are: Untreated, *n* = 4; RJx-01, *n* = 4. Muscle, blood, and urine samples were collected at sacrifice, and the numbers of animals sacrificed after 18 weeks of treatment are: Untreated, *n* = 10; RJx-01, *n* = 6.

The rationale for using the indicated concentrations (410 mg/kg bodyweight/d Met and 3.28 mg/kg bodyweight/d Gal) was derived from published studies to achieve beneficial pharmacodynamic effects in mice. Previous literature in mice showed beneficial effects of Met on some health parameters at a dose of 102.5 mg/kg/d, whereas 1,000 mg/kg/d was found to be toxic (39). We employed a higher dose than 102.5 mg/kg/d without having toxicity concerns, and a dose of 410 mg/kg bodyweight/d Met was selected to increase the likelihood of a benefit. The beneficial effects of Gal have previously been observed at a dose of 1 mg/kg/d administered through an osmotic pump (40). Because the compound is mixed directly into the food and to reduce concerns of inconsistent uptake in mice, the dose was adjusted to 3.28 mg/kg bodyweight/d Gal in mice.

Met HCl and Gal hydrogen bromide (HBr) were obtained from Farmhispania (Barcelona, Spain) and Fagron (Nazareth, Belgium), respectively.

### Body weight, food consumption, body composition, and metabolic assessment

#### Opa1^–/–^ mice and aged mice.

Body weight and food consumption were monitored. Measurements of lean and fat mass in live mice were determined using the EchoMRITM-100 (EchoMRI LLC).

#### Aged mice.

Mouse metabolic rate was assessed by indirect calorimetry using the PhenoMaster metabolic cages system (TSE). VO_2_, CO_2_, and EE levels were normalized to lean mass. In the aged mice, indirect calorimetry was performed after 10 weeks of treatment.

### Physical performance

#### Opa1^–/–^ mice.

The concentric training protocol consisted of the treadmill (Biological Instruments, LE 8710 Panlab Technology 2B) running to exhaustion, with no incline and a constant speed of 13 cm/s. Total running time was recorded for each mouse.

The in vivo muscle force is measured as previously described (41). Briefly, animals were deeply anesthetized, and the foot was mounted on a 305B muscle lever system (Aurora Scientific). The knee was blocked, and an electrical stimulation was applied to the sciatic nerve, inducing the isometric plantar flexion of the foot. The force-frequency curve was obtained by stimulating at increasing frequencies (starting with a single depolarization up to 150 Hz). Force was normalized to the weight of gastrocnemius and plantaris muscles to estimate specific force. Animals were then sacrificed by cervical dislocation according to the approved animal protocols, and muscles were dissected, weighed, and frozen. Experimental data were analyzed using a self-compiled program in LabView.

#### Aged mice.

At baseline and 18 weeks after treatment, mice performed concentric exercise on a treadmill (Biological Instruments, LE 8710 Panlab Technology 2B), as previously described (42). Total running time was recorded for each mouse.

#### Grip strength.

The grip strength was measured on forelimbs at baseline and 18 weeks after treatment using a commercially available Bioseb Grip Test device (Bioseb) as described previously (43).

### Histology

#### Opa1^–/–^ and aged mice.

The CSA was calculated by measuring the average cross-sectional analysis of all individual fibers from the entire muscle cross-section of gastrocnemius muscles based on an assembled mosaic image (at 10× magnification). The morphometric analyses were made using MATLAB Semi-Automatic Muscle Analysis using Segmentation of Histology (SMASH) software. For immunostaining, the antibody dystrophin (ab15277, Abcam) was used to identify the sarcolemma. Cryosections of the gastrocnemius muscles were stained for succinate dehydrogenase (SDH; S2378, MilliporeSigma). The number of glycolytic and oxidative fibers was calculated based on the SDH staining.

#### Opa1^–/–^ mice.

For immunostaining on frozen gastrocnemius slices, an antibody specific to NCAM (MilliporeSigma) was used. The secondary antibody, goat anti–rabbit Cy3 was obtained from Jackson ImmunoResearch (see Supplemental Table 2 for antibody information). Hoechst’s costaining allowed us to identify the subsarcolemmal position of myonuclei. Whole slices were also stained with WGA and shown as entire images.

#### Aged mice.

Paired box 7 (Pax7) staining was performed with frozen gastrocnemius slices fixed in PFA after cutting. Slices were treated with sodium citrate under boiling per 10 minutes for the unmasking procedure. Pax7 antibody (Developmental Studies Hybridoma Bank [DSHB], University of Iowa) was used. Total nuclei were stained with Hoechst, and muscle fibers were detected by the anti-dystrophin antibody. The secondary antibodies, Alexa Fluor 488 and Alexa Fluor 594, were obtained from Invitrogen. LAMP1 staining. For immunostaining, an antibody specific to LAMP1 (DSHB, University of Iowa) was used after fixing and permeabilization. The secondary antibody, Alexa Fluor 594, was obtained from Invitrogen. See Supplemental Table 2 for antibody information.

### Blood and urine measurements

#### Opa1^–/–^ mice.

After 90 days of treatment, plasma was obtained from blood collected from untreated *Opa1^–/–^* mice and mice treated with the individual compounds Met, Gal, and the combination drug RJx-01. The blood cytokines panel (Bio-Plex Pro Mouse Cytokine 23-plex Assay #M60009RDPD) was measured following the manufacturer’s instructions.

#### Aged mice.

After 18 weeks of treatment, mice were sacrificed, and urine and serum were collected from untreated and RJx-01–treated aged mice. Blood FGF21 (MilliporeSigma, EZRMFGF21-26K) and urine GDF15 (R&D Systems, MGD150) levels were determined following the manufacturer’s instructions.

### Quantitative PCR

#### Opa1^–/–^ and aged mice.

Total RNA was isolated from frozen tibialis muscle using TRIzol (Invitrogen), and complementary DNA was generated using the SuperScript III Reverse Transcriptase (Invitrogen) following the manufacturer’s instructions. Gene expression was determined by quantitative PCR (qPCR) as described previously (15). The primers are listed in Supplemental Table 1.

### Immunoblotting

#### Aged mice.

Muscles were lysed and immunoblotted as previously described (15). The membranes were visualized with the ImageQuant LAS 4000 and quantified using ImageJ software (NIH; https://imagej.nih.gov/ij/). Protein expression was normalized to GAPDH. A list of antibodies is provided in Supplemental Table 2.

### Transmission EM

#### Aged mice.

For EM, we used conventional fixation-embedding procedures based on glutaraldehyde-osmium fixation and Epon embedding. The number of severely damaged mitochondria was evaluated in micrographs of longitudinal sections taken at 13,700***×*** and 26,000***×*** magnification. Mitochondria with any or several of the following ultrastructural alterations were classified as severely damaged: presenting disruption of the external membrane, the presence of internal vacuolization, and/or disrupted internal cristae. The number of damaged mitochondria was quantified in a blinded fashion using ImageJ software. The visual inspections were conducted in a blinded manner as well. The same procedure was performed for the MVB quantification.

### NMJ morphology

#### Aged mice.

Whole-mount and immunofluorescence analysis of the NMJ was performed on extensor digitorum longus (EDL) muscles as described previously (44). NMJs exhibiting a clear overlap between pre- and postsynaptic structures were considered to be innervated while NMJs lacking an overlap were classified as noninnervated.

### Statistics

All data are presented as mean ± SEM. Survival analyses were performed using the Kaplan-Meier method, and the significance of differences between survival curves was calculated using the log-rank test corrected for multiple testing using the Benjamini-Hochberg method. Comparisons between 2 groups were done by 2-tailed Student *t* tests. To determine if there is a significant difference between more than 2 groups, a 1-way ANOVA was used. If the statistical difference was obtained, then a Holm step-down method, which uses an unpaired 2-tailed *t* test, was carried out to rank the different group comparisons by *P* value from smallest to largest. A linear mixed-effects model (LMEM) was used to fit the repeated measurement data and to compare the treatment groups. Animals were used as random in the LMEM. Outliers were detected by Grubbs. GraphPad Prism 8 (Statistical software) was used for all statistical analyses. *P* values below 0.05 were considered significant. To test for individual drug contribution, we used the higher single activity (HSA) model (45). According to this model, drug combinations are considered synergistic if the combinatorial effect is significantly larger than the largest effect of any of the single drugs. The numbers of animals in the different experiments varied for different reasons. In some instances, we analyzed only a subset of animals. First, animals were difficult to obtain in the case of aged or OPA1-deficient mice; thus, samples were not always available, due to the 28-month age requirement for the former and lethality of OPA1-KO animals or time necessary to induce OPA1 gene deletion. When samples were limited, the functional and morphological analyses were prioritized to address the effect on aging, and only the additional material was used for other measurements (e.g., GDF15, FGF21). Second, in situations involving assay complexity and associated time challenges (e.g., TEM, metabolic cages), only a limited number of samples were processed and analyzed. Third, in some instances, biological data were extremely homogeneous and required a reduced number of assays, while others required a greater collection. Fourth, mandatory ethical policies dictated that minimal numbers of animals be used to obtain significance. Fifth, in some experiments (e.g., morphology studies, histopathology, immunofluorescence), certain samples did not meet basic quality standards (e.g., artefacts due to freezing procedure that alters tissue morphology); therefore, they were excluded from the analysis (e.g., NCAM).

### Study approval

Animals were handled by specialized personnel under the control of inspectors of the Veterinary Service of the Local Sanitary Service (ASL 16, Padova, Italy), and the local officers of the Ministry of Health. Animal experiments were conducted according to the *Guide for the Care and Use of Laboratory Animals* (National Academies Press, 2011) as well as the Italian law for the welfare of animals. All animal experiments were approved by the Italian Ministero della Salute, Ufficio VI (Rome, Italy; authorization nos. 1060/2015 PR and 448/2021 PR).

### Data availability

Values for all data points in graphs are reported in the Supporting Data Values file.

## Author contributions

CT, ATJB, MS, and EMM conceived the study. CT, MS, and EMM developed the methodology. ATJB, MS, and EMM provided resources. CT, FIA, DA, LN, MB, SC, AZ, GM, and GF performed the experiments. CT, FIA, SR, BB, GD, ATJB, MS, and EMM performed the analysis. CT, MS, and EMM wrote the manuscript. MS and EMM visualized the data.

## Supplementary Material

Supplemental data

Supporting data values

## Version 1 08/08/2023

Electronic publication

## References

[1] Dent E (2018). International clinical practice guidelines for sarcopenia (ICFSR): screening, diagnosis and management. J Nutr Health Aging.

[2] Mijnarends DM (2016). Physical activity and incidence of sarcopenia: the population-based AGES-Reykjavik study. Age Ageing.

[3] Larsson L (2018). Sarcopenia: aging-related loss of muscle mass and function. Physiol Rev.

[4] Kulkarni AS (2020). Benefits of metformin in attenuating the hallmarks of aging. Cell Metab.

[5] Bannister CA (2014). Can people with type 2 diabetes live longer than those without? A comparison of mortality in people initiated with metformin or sulphonylurea monotherapy and matched, non-diabetic controls. Diabetes Obes Metab.

[6] Scott LJ, Goa KL (2000). Galantamine: a review of its use in Alzheimer’s disease. Drugs.

[7] Chang EH (2019). Cholinergic control of inflammation, metabolic dysfunction, and cognitive impairment in obesity-associated disorders: mechanisms and novel therapeutic opportunities. Front Neurosci.

[8] Hager K (2014). Effects of galantamine in a 2-year, randomized, placebo-controlled study in Alzheimer’s disease. Neuropsychiatr Dis Treat.

[9] Zong H (2002). AMP kinase is required for mitochondrial biogenesis in skeletal muscle in response to chronic energy deprivation. Proc Natl Acad Sci U S A.

[10] Bujak AL (2015). AMPK activation of muscle autophagy prevents fasting-induced hypoglycemia and myopathy during aging. Cell Metab.

[11] Mounier R (2013). AMPKα1 regulates macrophage skewing at the time of resolution of inflammation during skeletal muscle regeneration. Cell Metab.

[12] Sandri M (2010). MYTHO is a novel regulator of skeletal muscle autophagy and integrity. Nat Commun.

[13] Pavlov VA, Tracey KJ (2012). The vagus nerve and the inflammatory reflex--linking immunity and metabolism. Nat Rev Endocrinol.

[14] Lilienfeld S (2002). Galantamine--a novel cholinergic drug with a unique dual mode of action for the treatment of patients with Alzheimer’s disease. CNS Drug Rev.

[15] Tezze C (2017). Age-associated loss of OPA1 in muscle impacts muscle mass, metabolic homeostasis, systemic inflammation, and epithelial senescence. Cell Metab.

[16] O’Reilly GA (2014). Mindfulness-based interventions for obesity-related eating behaviours: a literature review. Obes Rev.

[17] Herndon LA (2002). Stochastic and genetic factors influence tissue-specific decline in ageing C. elegans. Nature.

[18] Cabreiro F (2013). Metformin retards aging in C. elegans by altering microbial folate and methionine metabolism. Cell.

[19] Akasaki Y (2014). Glycolytic fast-twitch muscle fiber restoration counters adverse age-related changes in body composition and metabolism. Aging Cell.

[20] López-Otín C (2013). The hallmarks of aging. Cell.

[21] Sousa-Santos AR, Amaral TF (2017). Differences in handgrip strength protocols to identify sarcopenia and frailty - a systematic review. BMC Geriatr.

[22] Deschenes MR (2010). Remodeling of the neuromuscular junction precedes sarcopenia related alterations in myofibers. Exp Gerontol.

[23] Dobrowolny G (2021). Age-related alterations at neuromuscular junction: role of oxidative stress and epigenetic modifications. Cells.

[24] Houtkooper RH (2011). The metabolic footprint of aging in mice. Sci Rep.

[25] Romanello V (2020). The interplay between mitochondrial morphology and myomitokines in aging sarcopenia. Int J Mol Sci.

[26] Conte M (2019). Human aging and longevity are characterized by high levels of mitokines. J Gerontol A Biol Sci Med Sci.

[27] Carnio S (2014). Autophagy impairment in muscle induces neuromuscular junction degeneration and precocious aging. Cell Rep.

[28] Klionsky DJ (2016). Guidelines for the use and interpretation of assays for monitoring autophagy (3rd edition). Autophagy.

[29] Hong J (2019). Lysosomal regulation of extracellular vesicle excretion during d-ribose-induced NLRP3 inflammasome activation in podocytes. Biochim Biophys Acta Mol Cell Res.

[30] García-Prat L (2016). Autophagy maintains stemness by preventing senescence. Nature.

[31] Verdijk LB (2014). Satellite cells in human skeletal muscle; from birth to old age. Age (Dordr).

[32] Sousa-Victor P, Muñoz-Cánoves P (2016). Regenerative decline of stem cells in sarcopenia. Mol Aspects Med.

[33] Blau HM (2015). The central role of muscle stem cells in regenerative failure with aging. Nat Med.

[34] Campagnola PJ (2002). Three-dimensional high-resolution second-harmonic generation imaging of endogenous structural proteins in biological tissues. Biophys J.

[35] De Haes W (2014). Metformin promotes lifespan through mitohormesis via the peroxiredoxin PRDX-2. Proc Natl Acad Sci U S A.

[36] Peymen K (2019). Myoinhibitory peptide signaling modulates aversive gustatory learning in Caenorhabditis elegans. PLoS Genet.

[37] Watteyne J (2020). Neuromedin U signaling regulates retrieval of learned salt avoidance in a C. elegans gustatory circuit. Nat Commun.

[38] Raizen D (2012). Methods for measuring pharyngeal behaviors. WormBook.

[39] Martin-Montalvo A (2013). Metformin improves healthspan and lifespan in mice. Nat Commun.

[40] Kakinuma Y (2014). Antimuscle atrophy effect of nicotine targets muscle satellite cells partly through an α7 nicotinic receptor in a murine hindlimb ischemia model. Transl Res.

[41] Blaauw B (2008). Akt activation prevents the force drop induced by eccentric contractions in dystrophin-deficient skeletal muscle. Hum Mol Genet.

[42] Lo Verso F (2014). Autophagy is not required to sustain exercise and PRKAA1/AMPK activity but is important to prevent mitochondrial damage during physical activity. Autophagy.

[43] Kang MJ (2022). Metformin induces muscle atrophy by transcriptional regulation of myostatin via HDAC6 and FoxO3a. J Cachexia Sarcopenia Muscle.

[44] Sartori R (2021). Perturbed BMP signaling and denervation promote muscle wasting in cancer cachexia. Sci Transl Med.

[45] Borisy AA (2003). Systematic discovery of multicomponent therapeutics. Proc Natl Acad Sci U S A.

