# Supplementary material for: A combination of metformin and galantamine exhibits synergistic benefits in the treatment of sarcopenia
Source: JCI Insight. 2023 Aug 8;8(15):e168787. doi: 10.1172/jci.insight.168787 (PMC10445681; doi:10.1172/jci.insight.168787)
Supplement: Supplemental data [file jciinsight-8-168787-s099.pdf]

A combination of metformin and galantamine exhibits synergistic benefits in the treatment of sarcopenia

Caterina Tezze[1,2], Francesco Ivan Amendolagine[1,2], Leonardo Nogara[1,2], Martina Baraldo[1,2], Stefano Ciciliot[1,2], Diletta Arcidiacono[3], Alice Zaramella[3], Giulio Masiero[1,2], Giulia Ferrarese[1,2], Stefano Realdon[3], Bert Blaauw[1,2], Giel Detienne[4], Ann TJ Beliën[4], Marco Sandri[1,2,5], Evi M Mercken[4].

[1]Department of Biomedical Sciences, University of Padova, via Ugo Bassi 58/b, 35121 Padova, Italy

[2]Veneto Institute of Molecular Medicine, via Orus 2, 35129 Padova, Italy

[3]Gastroenterology Unit, Veneto Institute of Oncology IOV-IRCCS, Via Gattamelata 64, 35128 Padova, Italy

[4]Rejuvenate Biomed, Wetenschapspark 13, 3590 Diepenbeek, Belgium

[5]Department of Medicine, McGill University, Montreal, QC H4A 3J1, Canada

Correspondence to: Marco Sandri, Veneto Institute of Molecular Medicine, via Orus 2, 35129 Padova, Italy. Phone: +39 0497923264, Email: marco.sandri@unipd.it and Evi Mercken, Rejuvenate Biomed, Wetenschapspark 13, 3590 Diepenbeek, Belgium. Email: evi@rejuvenatebiomed.com.

Supplemental Table 1: **Sequences of primers used for Quantitative Real Time Analysis**

	Forward	Reverse
GAPDH	CACCATCTTCCAGGAGCGAG	CCTTCTCCATGGTGGTGAAGAC
IL-1α	TCTCCTTCTCCTCCTTCTCC	GCTCCCTAAGTTCCCTGTCA
IL-1β	AAGGAGAACCAAGCAACGACAAAA	TGGGGAACTCTGCAGACTCAAACT
IL-6	TAGTCCTTCCTACCCCAATTTCC	TTGGTCCTTAGCCACTCCTTC
LC3	CACTGCTCTGTCTTGTGTAGGTTG	TCGTTGTGCCTTTATTAGTGCATC
P62	CCCAGTGTCTTGGCATTCTT	AGGGAAAGCAGAGGAAGCTC

Supplemental Table 2: Antibodies used in this study

Primary antibodies	Source (Identifier)	Dilution	Analysis
Mouse anti-GAPDH	Abcam (ab8245)	1:10000	WB
Rabbit anti-LC3B	Sigma (L7543)	1:1000	WB
Rabbit anti-P62	Sigma (P0067)	1:1000	WB
Rabbit anti-NCAM	Millipore (AB5032)	1:200	IF
Mouse anti-SV2 supernatant	Hybridoma Bank	1:100	IF
Mouse anti-2H3 supernatant	Hybridoma Bank	1:50	IF
Rat anti-LAMP1 (1D4B)	Hybridoma Bank	1:200	IF
Mouse anti-Pax7	Hybridoma Bank	1:50	IF
Rabbit anti-Dystrophin	Abcam (ab85302)	1:200	IF
Mouse anti-MHC IIB (BF-F3)	Hybridoma Bank	1:50	IF
Mouse anti-MHC IIA (sc-71)	Hybridoma Bank	1:100	IF
Mouse anti-MHC I (BA-D5)	Hybridoma Bank	1:50	IF
Bungarotoxyn, 594 conjugated	Invitrogen (b13423)	1:200	IF
WGA, 488 conjugated	Invitrogen (11570806)	1:100	IF

WB: western blotting; IF: immunofluorescence

Secondary antibodies	Source (Identifier)	Dilution	Analysis
Goat Anti-Mouse IgG (H + L)-HRP Conjugate (for GAPDH)	Biorad (1706516)	1:20000	WB

Goat Anti-Rabbit IgG (H + L)-HRP Conjugate (for LC3/P62)	Biorad (1706515)	1:2000	WB
Cy3 anti-rabbit IgG (for NCAM)	Jackson (111-165-003)	1:200	IF
Goat anti-Mouse IgG (H+L), Superclonal™ Recombinant, Alexa Fluor™ 488 (for SV2/2H3)	Invitrogen (A28175)	1:200	IF
Goat anti-Rat IgG (H+L) Cross-Adsorbed, Alexa Fluor™ 594 (for LAMP1)	Invitrogen (A-11007)	1:200	IF
Goat anti-Mouse IgG (H+L) Cross-Adsorbed Secondary Antibody, Alexa Fluor™ 594 (for Pax7)	Invitrogen (A-11005)	1:200	IF
Alexa fluor 488 anti-rabbit (for dystrophin in the Pax7 immunostaining)	Jackson (111-145-144)	1:200	IF
Alexa Fluor 405 goat anti-mouse IgG2b (MHC I)	Jackson ImmunoResearch Europe (115-475-207)	1:200	IF
Alexa Fluor 448 goat anti-mouse IgG1 (MHC IIa)	Jackson ImmunoResearch Europe (115-545-205)	1:200	IF
Alexa Fluor 594 goat anti-mouse IgM (Cy3) (MHC IIb)	Jackson ImmunoResearch Europe (115-505-020)	1:200	IF
Alexa Fluor 647 goat anti-rabbit IgG (dystrophin)	Jackson ImmunoResearch Europe (111-607-003)	1:200	IF

Supplemental Table 3: Significant synergy between metformin and galantamine was detected in terms of adult *C. elegans* survival

**Condition**	**[Met] (mM)**	**[Gal] (µM)**	**Mean lifespan *vs* untreated control**	**Mean lifespan *vs* Met 25 + Gal 100**
Untreated control	0	0		-18.1%***
Met 25	25	0	+11.9%***	-8.3%*
Gal 100	0	100	+6.8%*	-12.5%***
Met 25 + Gal 100	25	100	+22.1%***	

Met: metformin; Gal: galantamine; $^{*}p_{\text{log-rank}} < 0.05$, $^{***}p_{\text{log-rank}} < 0.001$.

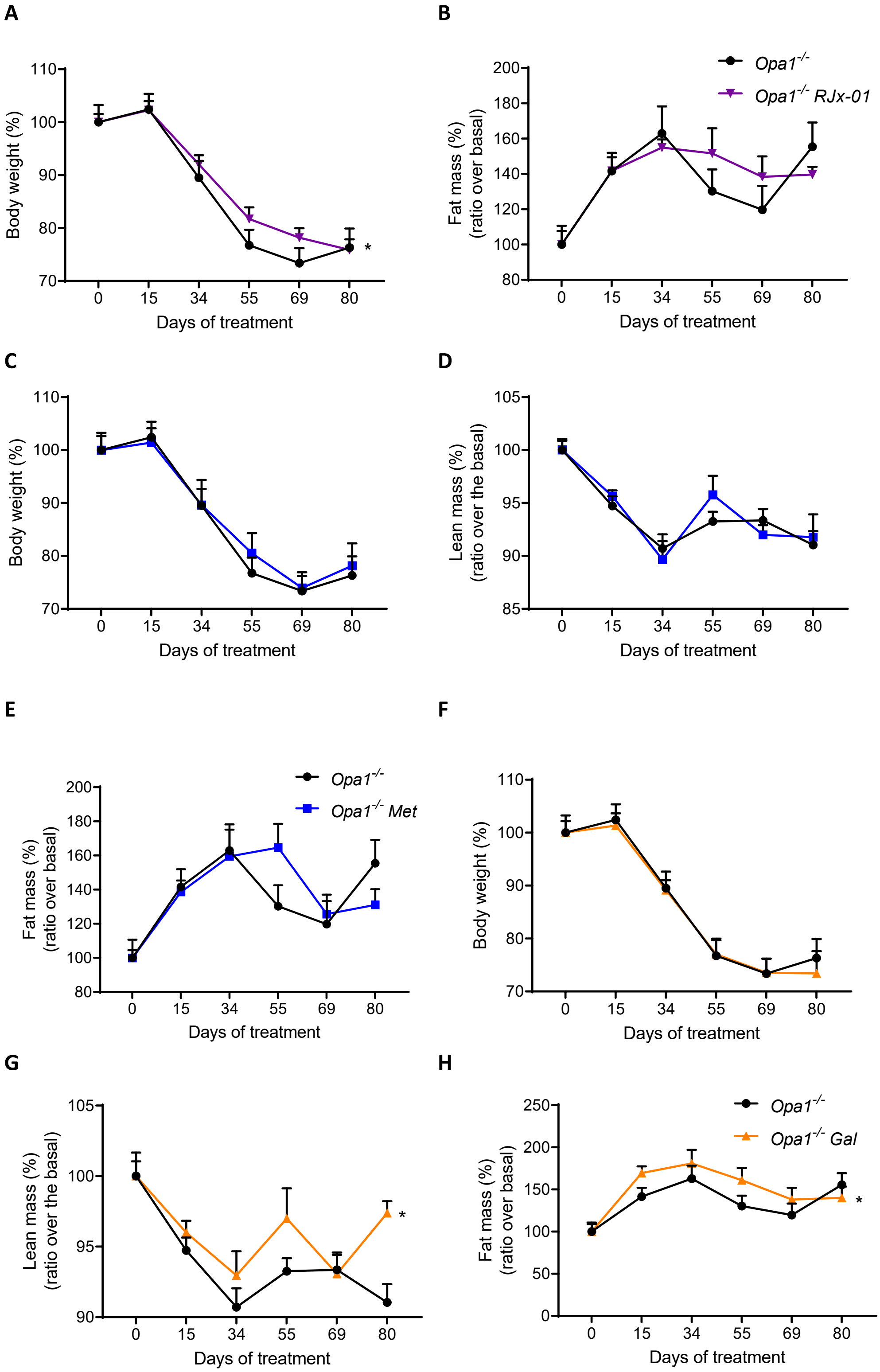
A
Body weight (%)
110
100
90
80
70
0 15 34 55 69 80
Days of treatment
*
B
Opa1-/-
Opa1-/- RJx-01
Fat mass (%)
(ratio over basal)
200
180
160
140
120
100
80
0 15 34 55 69 80
Days of treatment
C
Body weight (%)
110
100
90
80
70
0 15 34 55 69 80
Days of treatment
D
Lean mass (%)
(ratio over the basal)
105
100
95
90
85
0 15 34 55 69 80
Days of treatment
E
Opa1-/-
Opa1-/- Met
Fat mass (%)
(ratio over basal)
200
180
160
140
120
100
80
0 15 34 55 69 80
Days of treatment
F
Body weight (%)
110
100
90
80
70
0 15 34 55 69 80
Days of treatment
G
Lean mass (%)
(ratio over the basal)
105
100
95
90
0 15 34 55 69 80
Days of treatment
*
H
Opa1-/-
Opa1-/- Gal
Fat mass (%)
(ratio over basal)
250
200
150
100
50
0
0 15 34 55 69 80
Days of treatment
*

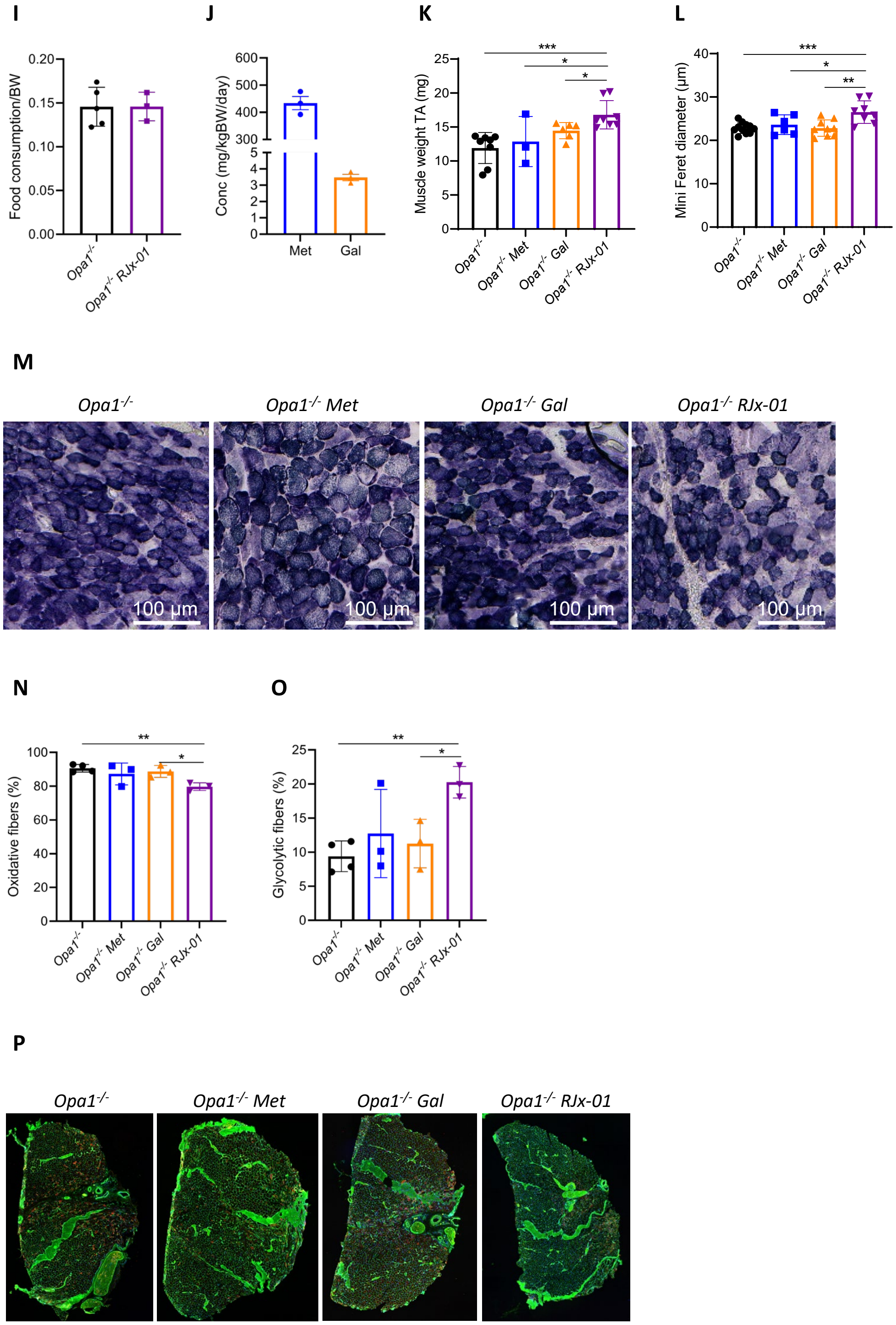

I
Food consumption/BW
0.20
0.15
0.10
0.05
0.00
Opa1-/-
Opa1-/- RJx-01
J
Conc (mg/kgBW/day)
600
500
400
300
5
4
3
2
1
0
Met
Gal
K
Muscle weight TA (mg)
25
20
15
10
5
0
***
*
*
Opa1-/-
Opa1-/- Met
Opa1-/- Gal
Opa1-/- RJx-01
L
Mini Feret diameter (µm)
40
30
20
10
0
***
*
**
Opa1-/-
Opa1-/- Met
Opa1-/- Gal
Opa1-/- RJx-01
M
Opa1-/-
Opa1-/- Met
Opa1-/- Gal
Opa1-/- RJx-01
100 µm
100 µm
100 µm
100 µm
N
Oxidative fibers (%)
100
80
60
40
20
0
**
*
Opa1-/-
Opa1-/- Met
Opa1-/- Gal
Opa1-/- RJx-01
O
Glycolytic fibers (%)
25
20
15
10
5
0
**
*
Opa1-/-
Opa1-/- Met
Opa1-/- Gal
Opa1-/- RJx-01
P
Opa1-/-
Opa1-/- Met
Opa1-/- Gal
Opa1-/- RJx-01

Figure S1. (A-B) Percentage of body weight and fat mass of *Opa1*$^{-/-}$ mice fed a diet supplemented with RJx-01. (*Opa1*$^{-/-}$*:* n=11*, Opa1*$^{-/-}$ *RJx-01:* n=8*).* **(C-H)** Percentage of body weight, lean mass and fat mass of *Opa1*$^{-/-}$ mice fed a diet supplemented with metformin (*Opa1*$^{-/-}$*:* n=11*, Opa1*$^{-/-}$ *Met:* n=6*)* or **(F-H)** galantamine (*Opa1*$^{-/-}$*:* n=11*, Opa1*$^{-/-}$ *Gal:* n=6*).* **(I)** Food consumption over bodyweight and **(J)** dosages of metformin and galantamine per kg of bodyweight (*Opa1*$^{-/-}$*:* n=5*, Opa1*$^{-/-}$ *RJx-01:* n=3*)* **(K)** Weights of tibialis anterior muscles (TA) (*Opa1*$^{-/-}$*:* n=8*, Opa1*$^{-/-}$ *Met:* n=3*, Opa1*$^{-/-}$ *Gal:* n=6*, Opa1*$^{-/-}$ *RJx-01:* n=8*)* and **(L)** minimal Feret diameter of transversal gastrocnemius sections (*Opa1*$^{-/-}$*:* n=11*, Opa1*$^{-/-}$ *Met:* n=6*, Opa1*$^{-/-}$ *Gal:* n=9, *Opa1*$^{-/-}$ *RJx-01:* n=8*)* **(M)** SDH staining (Scale bar = 100 µm) and quantification of the number of **(N)** Oxidative, and **(O)** Glycolytic fibers in the different conditions (*Opa1*$^{-/-}$*:* n=4*, Opa1*$^{-/-}$ *Met:* n=3*, Opa1*$^{-/-}$ *Gal:* n=3*, Opa1*$^{-/-}$ *RJx-01:* n=3*).* **(P)** Representative muscles stained with NCAM and WGA. Data are mean ± SEM. *$P < 0.05$, **$P < 0.01$, ***$P < 0.001$. In panel **A, B, C, D, E, F, G** and **H** a linear mixed-effects model was used, a two-tailed Student's *t* test was performed in panel **I** and **J**, and 1-way ANOVA followed by Holm step-down method (two-tailed Student's *t* test) was performed in panels **O** and **P**.

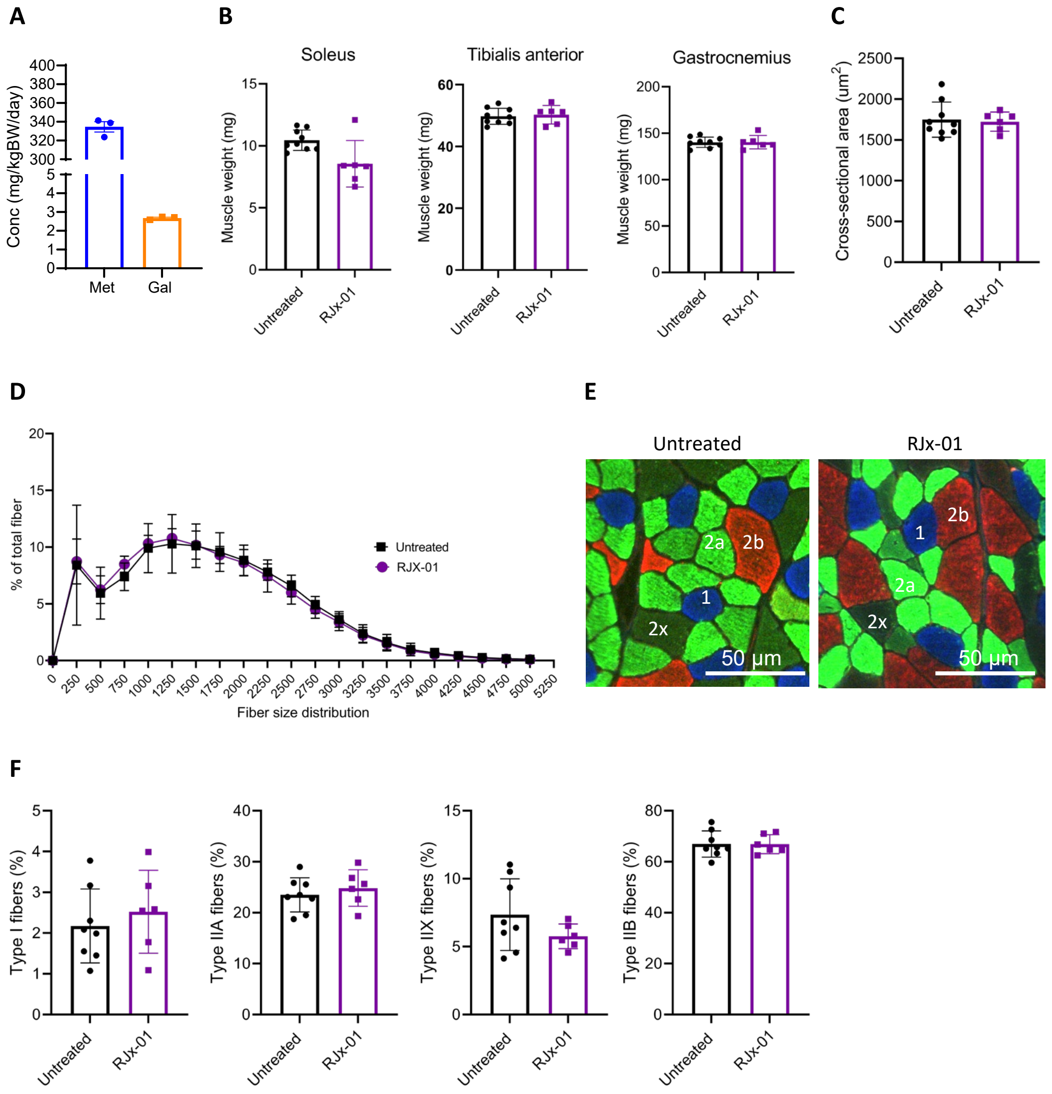

Figure S2. **(A)** Dosages of metformin (Met) and galantamine (Gal) per kg of bodyweight in aged mice (n=3). **(B)** Weights of muscles (soleus, tibialis anterior, gastrocnemius) of aged mice fed a control diet or a control diet supplemented with RJx-01. **(C)** Myofibers cross-sectional area analysis of gastrocnemius muscle, and **(D)** the relative fiber size distribution. **(E)** Representative images of the fiber type staining (Scale bar = 50 µm) and **(F)** The number of fibers (Type I, IIA, IIX, IIB) expressed as a percentage (n= 5300-7500 fibers per muscle). (Untreated: n=9; RJx-01: n=6). Data are mean ± SEM.

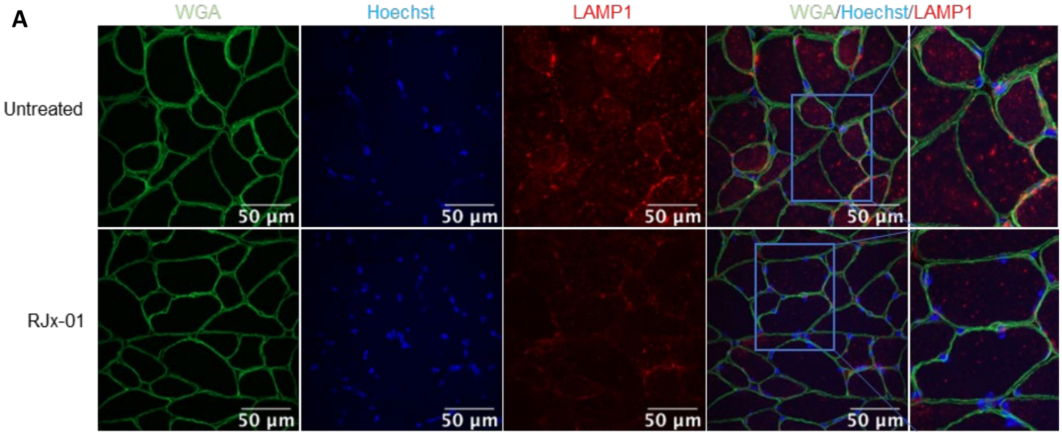

Figure S3. **(A)** Representative images of LAMP1 expression after 18 weeks of treatment. (Untreated: n=3; RJx-01: n=6). Scale bar = 50 μm.
